# Music education contributes to development and personal change in young adults with disabilities

**DOI:** 10.3389/fresc.2022.1046480

**Published:** 2022-12-23

**Authors:** Cristina Lundqvist-Persson, Gärd Holmqvist

**Affiliations:** The Skaraborg Institute for Research and Development, Skövde, Sweden

**Keywords:** disability, emotional wellbeing, emotional and social development, music training, learning

## Abstract

In Sweden as in many other countries, there has been increasing recognition of the importance of health, social participation, and active leisure time for people with disabilities. Against this background, a three-year music education was started for a group of young adults with disabilities in order to enhance their wellbeing, learning, and emotional and social development. The aim of the study was to evaluate the results of a 3-year education program with set goals for young adults with disabilities using a qualitative method. The study was conducted from autumn 2014 to 2018. Four semi-structured interviews were conducted with the participants, the first at the beginning of the education, after the first year, second, and third year respectively. The interviews were tape recorded and transcribed verbatim. The teachers and care staff made process notes about the development of each participant. The transcribed interviews and process notes were analyzed using Thematic Content Analysis. The education achieved its purpose and goals as evidenced by participants, teachers, and staff. It showed that music education training may revitalize people with disabilities. Furthermore, it demonstrated that persons with disabilities can learn, develop, and even change on a personal level, if they are given the right conditions.

## Introduction

The importance of health, social participation, and active leisure time for people with disabilities has been increasingly recognized in Sweden. One of the reasons is the UN Convention on the Rights of Persons with Disabilities (CRPD), the international human rights treaty of the United Nations (1) intended to protect the rights and dignity of persons with disabilities. The convention has been in force in Sweden since 2009, which means that all laws and public activities in Sweden must comply with the articles of the Convention. In Sweden people with disabilities receive help and support in accordance with their needs as set out in the Law on Support and Service for disabled people (LSS) or The Social Services Act (SoL) (2). According to The National Board of Health and Welfare (3), there are 75,000 persons in Sweden covered by the LSS.

Another reason is that the Swedish Public Health Agency has reported that people with disabilities continue to have poorer living conditions, lifestyles, and health compared with those without a disability. People with disabilities more often complained about lack of meaningful employment, lack of well-being, and were socially isolated (4).

Human health has been increasingly affected by lifestyle and life situation (4). However, individuals cannot always influence their lifestyle and life situation to achieve health, as it may be due to societal factors (5). Therefore, in 1991 the World Health Organization (WHO) proposed that society should offer so-called “supportive environments” to promote existential health and well-being. Creative and cultural activities can be considered such a “supportive environment” (6).

The importance of culture, especially music, for health has received increasing attention in society. It has been shown that creativity and music have an impact on the quality of later life (7), that music training during childhood induces changes in brain structure (8), that music therapy has a positive effect on pain, anxiety, and depression in patients (9), and that music is good for public health (10). Music and musical activities with different theoretical backgrounds have been used in various forms of therapy. For example, analytically oriented music therapy has its basis in psychoanalysis, but also in communication theories, interaction theories, and development theories (11). Another form of therapy is creative music therapy, which was developed by the American composer and pianist Paul Nordoff, together with the English special education teacher Clive Robbins (12). This therapy was inspired by the anthroposophical view of disability, which is that humans have a healthy core and can develop irrespective of disability. Nordoff and Robbins (12) were influenced by humanistic psychology and Maslow's theories about man's realization of his full potential (13). Function-oriented music therapy was started in Sweden by the music therapist Lasse Hjelm (14) and influenced by neuropsychology, neurophysiology, and developmental psychology. It is a non-verbal method mainly used in habilitation, rehabilitation, and school.

It was against this background that a music training program called Music Passion was initiated. The number of post-secondary educations in Sweden is limited for people with disabilities and universities do not exist for them. Therefore, it was important to start an education that differs from the daily activities offered to this group.

Together with a post-secondary music school, the Social Services in a small town in Sweden decided to start a three-year music education (Music Passion) for young adults with disabilities. The school employed music teachers who had experience of working with people with disabilities, and they designed a new music training program adapted to persons with varying degrees of intellectual disability and different additional disabilities.

Because the training was unique in Sweden and largely funded by a research foundation, evaluation by researchers outside the project was required. Thus, the authors were not involved in the construction of the training program and had never met the participants before.

As far as we know, there are no such studies but more studies about music as a therapeutic tool.

### Evaluation

#### The aim

The aim of the study was to evaluate the results of a 3-year education program with set goals for young adults with disabilities using a qualitative method.

#### Description of the training program

Music Passion involved eight hours of theatre and dance per week and a final production in the form of a performance at the end of each year. Two teachers carried out the training at the music school supported by technical staff from the school and staff involved in the care of the participants. The care staff members were given opportunities to familiarize themselves with the training and its progress so that they could follow up the different elements at the home of the participants.

The performance at the end of each academic year had an audience. All three performances were well attended by people in the town.

The training activities were inclusive, which means that participants with different interests and conditions were able to participate. Although the education was based on the individual's resources rather than on the disability, the teaching was nevertheless adapted to the target group's functional deficiencies. Shortly after the start of the intervention the participants were divided into two groups based on their functional level in certain elements of the training.

The education had a preliminary plan and structure but was further developed during the intervention. The goals of the training were that upon completion, the participants would have learnt one or more musical skills (Table 1), developed the ability to collaborate based on music and performance, experience increased confidence and safety when part of a group, and have had a role on or next to the stage in a production.

**Table 1:** Elements included in the education.

Instrument
Develop interest in an instrument you already know to some extent.
Opportunity to get to know a new instrument
Singing
Practice different ways of performing a song; playback, singback, solo, choir, with accompaniment
Ensemble (Collaboration)
Practice playing and singing with others in small groups and “bands”.
Practice your role in relation to the others in the group.
Repeat a piece of music in preparation for a performance
Create using software
Individually work with software for music creation.
Create music/compose
Create your own music based on a given background or beginning
Write lyrics to existing or newly composed music
Acting and playing a role
Stage presence and audience contact
Perform individually and in groups
Microphone technology

An additional goal consisted of expected synergy effects such as increased positive self-esteem, greater self-confidence, improved mental health and well-being.

The education was internship oriented, which means that learning takes place through practical exercises and activities.

### Ethical approval

Ethical principles were followed in accordance with the World Medical Association's Declaration of Helsinki (15) and the Swedish Research Council's national guidelines (16). Extra sensitivity and accuracy were considered important as the participants may not fully understand what it means to participate in a research study.

The evaluation research plan was therefore thoroughly reviewed with the participants, informed about the different parts of the evaluation. If the person seemed unable to fully understand the implications of participating, her/his close relative or accompanying care staff member signed the consent form.

We were careful to emphasize that the participants were free to participate in the education even if they did not wish to take part in the evaluation of it. Furthermore, that they could cancel their participation at any time without giving a reason. This information was given not only to the participants but also to the teachers, care staff members, and relatives. As the participants may have difficulty expressing their opinion and wishes and might feel insecure with people they do not know, they only had contact with one of the authors and were accompanied by a care staff member or relative during the interviews. Both authors have years of experience of contact and communicating with people with disabilities.

The study was approved by the Regional Ethical Review Board in Gothenburg, Sweden (Dno: 686-14).

### Theoretical frame of references

The Music Passion training program has a social pedagogical theoretical perspective (17). Social pedagogy includes strategies for creating conditions for community and communication, which can support the individual in her/his quest for an active life together with others. This is in accordance with Vygotsky's sociocultural theory of human learning (18), which describes learning as a social process and the origin of human intelligence in society and culture. The major theme of his theoretical framework is that social interaction plays a fundamental role in the development of cognition and learning. The Music Passion training provides opportunities for experiences and activities that include both communication and social interaction. When social participation is created, it has an intellectual and emotional impact that can positively affect/influence an individual's self-esteem.

The evaluation of Music Passion was based on human development theory, including emotional, social, and cognitive development in relation to young adults with a disability. Daniel Stern (19), who focused on the relational aspect of human emotional development, emphasized the importance of intersubjective communication, namely *the sharing of subjective experience between two or more people,* for the development of the self. The Music Passion training program made such communication with others possible, thus strengthening the self and promoting health.

The importance of attachment and trust in relationships is well known since Bowlby (20) described it in the Attachment, Separation, and Loss trilogy. In Music Passion attachment was crucial for individual learning and development. Attachment involved feeling safe and having the courage to express oneself through music, acting, and performing. Peter Fonagy and Anthony Bateman (21) further expanded the attachment theory and developed the concept of mentalizing, the ability to understand oneself and others, which takes place within the context of the attachment relationship. In Music Passion, the quality of the teachers' relationship with the participants, as well as the relationship between the participants, were of paramount importance.

There is consensus that motivation is important for learning. In the so-called Self-Determination Theory (SDT) (22), Deci and Ryan distinguish between different types of motivation and in 2000 shed further light on the distinction between intrinsic and extrinsic motivation and their different meanings in learning (23). Therefore, how the participants in Music Passion expressed their motivation for participation and learning was of great interest.

Stern (24) stressed the importance of vitality affects for human development and health, defined as an intrapsychic feeling of energy evoked in the encounter with dynamic and stimulating events. In his book Forms of Vitality (24), he explored vitality forms in music, dance, theatre, and cinema, which can contribute to the understanding of the impact music may have on people. Koel (25) stated that music affects the brain and evokes emotions. His study is in accordance with Hyde et al. (26), who found that musical training shapes structural brain development.

## Materials and methods

### Research questions

The research questions were formulated based on the goals of the education.

These were:

• To what extent did the participants gain new knowledge in a specific area of interest?
• To what extent did the participants develop increased functional collaboration with their fellow participants?
• To what extent did the participants develop a more active lifestyle, and did they experience meaningful activities?
• To what extent did the participants experience increased social participation and engagement, stronger positive self-esteem, self-confidence, improved mental health and well-being?

### Participants

Seventy-two young adults with mainly intellectual disabilities were invited to participate in this music education. All of them had previously shown an interest in music and many of them had reported that they were somewhat dissatisfied with life. All those invited had help and support in accordance with their needs as set out in the Law on Support and Service for disabled people (LSS) or The Social Services Act (SoL) (2, 27).

The seventeen persons (10 women and 7 men) who registered their interest in participating in the education were accepted. They were also willing to be part of the research project, about which they were informed together with a close relative or care staff member. After the provision of verbal information, they received written information about the research project before signing their informed consent.

Of the 17 participants who started the music education, three dropped out, one at the beginning due to a difficult social situation, the second after a year because he wanted more individual training and the third after the second year because he had obtained employment. Three other participants were added, one at the start of the second year and two at the start of the third year. In total, 20 persons were thus involved, 14 for the entire 3 years, and data from these 14 participants is included in the analysis. The background factors are presented in Table 2.

**Table 2:** Participants involved for the entire 3 years training and their background variables.

Backgrounds variables	Women (9)	Men (5)
Mean Age (years)	29	32
Mild intellectual disability	6	5
Severe intellectual disability	3	1
Autism	1	0
Down Syndrome	2	1
Cerebral Pares	1	1
Additional disabilities hearing, visual, speech	3	3
Compulsory School for pupils with learning disabilities	6	5
Compulsory School for children with severe learning disabilities	3	1
Accommodation		
Group accommodation with staff	3	2
Own apartment with personal assistance support	3	3
Own apartment supported by staff for certain activities	2	1
Parent's Home	1	0
Daily work or activity duration from 6 to 35 h a week	9	5

### Data collection

#### Interviews

As a qualitative researcher, one is part of the research process, which will be influenced by the researcher's previous experiences, assumptions, and beliefs*.* There is always a risk that the findings are a result of what the researcher wishes and/or has set out to find.

Furthermore, there is the inherent pitfalls of power differentials between participants and the researcher. The researcher is an expert who will determine the results of the study, while the participant is asked to give something of her/himself, often without any control over the outcome. In this study, this was extra sensitive considering that the participants were people with intellectual disabilities.

Given the above risks of bias we tried to avoid these problems by not being involved in the construction of the training program. Moreover, we had no previous contact with the teachers or participants, and we chose the data collection and analysis method with care. Both of us researchers were involved in the data analysis.

Four semi-structured interviews with follow-up questions were conducted with each participant. They took place at the beginning of the participants' training, after the first year, second year, and third year (28). Under supervision of the first author (CLP) the first three interviews were conducted by a person known to the participants from the Social Services in the participant's home. The fourth interview took place at the music school and was conducted by the first author.

It is not always easy for people with disabilities to express what they feel, think, want, and reflect on. The participants were therefore encouraged to bring a close relative, or care staff member to the interview, which everyone did. The statements made by the relative or staff member were confirmed by the participant, who agreed with what was said by nodding, gesturing and/or facial expressions. To enhance understanding the participants were encouraged to give examples of what they reported.

At the first interview the participants were asked to talk about themselves, areas of interest, leisure activities, social network, expectations of the upcoming education, if they wanted to change something in their life, and why they applied for the training. They were also asked if they have chosen it themselves or if someone else had chosen for them. The aim was to obtain a description of their person, their personal situation, and how they felt.

The other two interviews mainly focused on the training in relation to the goals and research questions. The fourth interview was a summary of what they had learnt, what the training had meant to them, how it had affected them, what had been good and less good but also about collaborating with others and social relationships.

*All the interviews were audio recorded and transcribed verbatim*.

#### Process notes

The teachers and care staff made process notes about the development of the individual participant and group. They noted if the participant learned something new, expressed something special about her/himself, had been especially satisfied or dissatisfied with an activity in the training, had made progress or exhibited changed behavior, e.g., became more open and social or withdrawn or more engaged and active. At the end of each year the staff summarized these process notes and handed the summary over to the researchers.

To make the best possible evaluation of the education, each participant's attendance at the lessons was registered throughout the training.

### Data analysis

All the recorded interviews were transcribed by a research secretary. The transcribed interviews and the process notes from the educators and staff were analyzed using thematic content analysis, which “offers a really useful qualitative approach for those doing more *applied* research” (29, 30). In this study the aim was to discover the participants' perception, experiences, feelings, thoughts, and changes related to the training. Thematic content analysis was used because it is a flexible research tool that can provide a potentially rich and detailed description based on what is classified as big and complex data. Both a deductive and an inductive analysis is possible as well as an analysis on a semantic and latent level. These different levels of analysis can be used simultaneously (29, 30).

Thematic analysis involves constantly moving back and forth between the data and the themes and is carried out in several steps.

In the present study, the interviews and process notes were first read to become familiar with the material. The material was then carefully reviewed and interesting features in the data were coded in a systematic way using a deductive semantic approach based on the formulated research questions. We focused on how the participants spoke about their experiences, what they had learnt, their views on collaboration and becoming involved in the musical activities. The analysis also included what the teachers and staff had written about these aspects. Relevant data for each code was then collated.

When all data had been coded and collated, we re-focused the analysis at a broader level of themes, sorting the different codes into potential themes and checked if the themes worked in relation to the coded extracts and the entire data set.

The data was also reviewed using an inductive semantic approach to see if more themes emerged. All results are presented in themes and categories. (See the Result section below).

We conducted the data analysis first separately and then together. The assigned codes and themes largely agreed and after discussion and further reflection interrater agreement could be achieved.

## Results

The quotations presented in the results are statements from participants, teachers, and staff. Statements made by staff or relatives due to the participants’ difficulties expressing themselves have been slightly edited but the content reflects what was said.

In general, the attendance at the training was very high, except for one participant who was ill for long periods of time and hospitalized. In the first year, participants’ attendance was 97%, the second year 95%, and in the third year also 95%. The high attendance was probably due to the fact that the participants really enjoyed attending the classes. At the last interview one staff member said to the participant: *“You’ve never had a rough morning when you were going to Music Passion, you always jumped out of bed”*.

### Themes

The analysis resulted in the following themes: *New skills, Social and personal development, The meaning of music and Music Passion, and Staff reflections on participants*’ *changes* with associated categories.

### New skills

Achieving new skills means that the participant developed a previous music skill or learned something new. Three categories emerged: *Playing an instrument, Singing, and Acting*.

In the interviews, there was no doubt that the teaching *per se* was perceived as meaningful: *“Drums are my life, I've become a little better as a drummer”* and that the training was taken seriously: *“It's not just a thing you do, you learn something”.* When participants discovered new talents, it was a surprise for everyone: “*I have learnt a lot, I was surprised to have learnt so much”.* Another participant said: “*I was surprised that I could learn so much by heart”*.

#### Playing an instrument

Several participants already had experience of playing an instrument, but they developed their playing by using all the bass strings instead of playing entire songs on the same string, even playing A, or playing “*terse chords”* on the keyboard instead of just one note at a time. The staff process notes contained many comments about how the participants developed their playing skills: “*The student has started playing terse chords instead of basic tones”* or *“The student has learned to play accords in minor”*.

Other participants appreciated the new experience of learning to play an instrument: “*I have learnt new things in music and theory”*. One participant confirmed the following suggestion from the relative by nodding and giving a little smile: “*You've learned to drum whole songs and feel the beat, when to start and finish the songs, is that right?”.* Developing motor skills proved to be important: “*Yes, I learned to play bass, but more needs to be done with my fingers”.* Staff members confirmed that the development of motor skills supported new music skills: “*The student has played two congas and used his left and right hand at the same time” or “The student can now better control the drumming and motor skills”*.

#### Singing

According to the staff members, the development of singing skill could concern the participants finding their own voice: “*The participants have developed their singing voice”*. Learning new singing skills also included learning new songs: *“I learned a lot of new songs, yes”* and learning texts: *“The student has learned song lyrics very quickly”* or singing whole songs and becoming more secure about their melodies: “*I have made a lot of progress; I have gotten better at almost everything. For example, I sing the song better”.* Playing an instrument and singing were skills that were close to each other: “*The participant played the drums and sang at the same time, which was his goal”*.

#### Acting

Developing acting skills was an ongoing process over the three years and the participants were able to perceive that they had developed: “*I had a smaller role in the first performance and then a bigger role in the second”.* They developed a stage language or microphone technique by singing solo on stage. Acting also included improvisation: “*They have shown that they can now improvise”* as well as memorizing lyrics: *“They can now memorize lines and lyrics by heart and perform them in public”.* Creating characters to fit the songs was a newly developed skill for one participant: “*The student has learned to create characters for the songs*”. In general, it was important to learn skills before the performance: *“They have become more and more involved, especially during the theatre and dance lessons”.* Some participants exhibited surprising skills: “*The student has written her own reply”* and for one student theatre turned out to be the real thing: *“The student creates made-up characters for all songs, acts well and likes to dance”*.

### Social development

The sub-themes of social development describe the experience of being in a group, interacting with others, participating in activities, and changed behavior. Three categories were developed: *Group experience and feelings, Group interaction, and Changed behavior*.

#### Group experience and feelings

The participants gained a positive feeling from being in a group together and started to see the needs of others: “*They realized that they may constitute a support for others who find things difficult*” and made sure that all the other participants were doing well. Some participants became mentors for others who needed support. One participant said: *“The more time passed, I felt I had started making friends and then there was contact with everyone”**,*** and self-confidence increased: *“Then, when you got to know everyone, self-confidence grew”.* The group feeling counteracted loneliness: “*When I come to Music Passion and meet friends I don't feel alone”*.

Another important positive experience was participating in the new community and having something to refer to in other contexts: “*A community has developed, and you have something to refer to when you meet outside the training”.* One parent stated: “*The participants feel that they are seen, they appreciate what they have done together and the fact that they have friends to say hello to in town means a lot”.* The participant listened and nodded in agreement.

#### Group interaction

At the beginning, participating in the different group activities was a bit scary: *“I was a little nervous at first, but then it disappeared”.* When they felt more secure, they became more communicative and actively sought contact with others: “*Nowadays you can see that they often stop at the end of the day and talk to each other”.* Some students who had speech impediments started to use more signs to increase communication and even participants with major disabilities were integrated: “*Through joint activities, there has been integration in the group even for participants with major disabilities”.* The participants gradually became more involved in the education and in decision making. For example, when preparing the script for the third-year performance they “*came up with many good ideas”.* Music Passion initiated new relationships: “*You get to know the others better and better, we can joke with each other, hug and be appreciated”,* social life expanded, and their daily life outside Music Passion changed: *“These people knew each other before Music Passion but never socialized in their spare time”*.

#### Changed behavior

Over time all participants increased their ability to collaborate: “*They have gained a greater sense of the group and their collaboration with the other participants has developed a lot”.* They matured during the training and became more emotionally aware: “*They have overcome their difficulties and matured and can manage their feelings, thus the group feeling increased”*. Some had developed a more open form of communication: “*Now you can see even shy participants putting their hand on a fellow participant's shoulder and talking to the others”*.

The participants themselves became aware that they had learned to play music with others: “*I've learned to play the bass and play alongside others, everyone has taken big steps since we started”.* Over time the students gladly came up with their own ideas and suggestions and were proud about their participation in Music Passion: “*He is very proud of his performance in the show”*.

Some of the participants were relatively active even before the education, but those who were less active in their spare time had increasingly started to participate in several different activities: “*It's fun to have some people you can visit when you go out”.* The participants talked more with staff and fellow participants and were more involved in the activities. In the interview one parent stated: *“You like being in this group and now you know how to behave, otherwise you're extremely quiet”* and the participant nodded in agreement.

A stronger group feeling could also manifest itself in increased ability to adapt: “*The students have learned that they cannot always do what they think is most fun, they must do things in order for the group to improve”.* A couple of the participants had broken the isolation they were in: “*I've made friends, I now have more friends”* and two of them had developed a relationship and moved together into their own home: “*So we started hanging out a lot outside of the music training, we got closer together”.* Both proudly showed off their engagement ring at the individual interview.

### Personal development and change

The Music Passion education was perceived by the participants as very positive and had changed them and their daily life in many ways. Two categories emerged: *Personal inner change* and *Consequences of inner change*.

#### Personal inner change

The participants experienced a huge difference compared with their life before Music Passion. One participant experienced inner change as having become a different person: “*I've undergone great development as a person, so that who I am today is not the same as before”.* Another said: “*I gained confidence; it gets stronger and stronger”.* Teachers and staff also noted the changes on the psychological level and stated: “*We see a positive development in all participants, both individually and as a group”*. In the interviews, several of the participants talked about a difficult childhood and adolescence, especially in school and other social contexts. This resulted in negative self-esteem, low self-confidence**,** and in some cases even social isolation: “*I used to feel very bad, there's been a bit of bullying, it wasn't funny, it's not like that now, now I'm fine”.* Some participants gained insight into their own inner changes and were pleased to see their own development: *“Feels good because I think it's fun to see a development in myself”*. Some had experienced that they could develop regardless their disability and had discovered new sides of themselves: “*So regardless of diagnoses or other type of disability, I have proved I am able, even though I have some difficulties”*.

#### Consequences of inner change

As inner change led to a greater sense of security it helped the participants to take the initiative: “*I have learned and dare to take initiatives, dare to try again”.* One participant said*: “I've become more open and talk and my shyness has disappeared”.* Increased openness and tolerance were other consequences of inner changes in the participants: “*They have become more tolerant in messy situations, are involved in all activities”*. Another consequence was less rigidity and increased flexibility: “*They have become more flexible, are more outgoing, talk more to others and have become more aware of their own voice”.* The participants were more independent: “*They have become more independent both in the lessons and in their everyday life”* and *“They are more aware of their own abilities and limitations”.* All the participants experienced an increased sense of well-being compared with before the intervention: “*I feel good because I'm doing more things now”.* The teachers noted that some participants had even overcome health problems: “*They want to participate in the training despite health problems”*.

### The impact of music passion and music

The meaning of music and Music Passion can be described by their impact on the participants, who stated that they were stimulating, engaging, and linked to an activity. Two categories emerged: *The impact of Music Passion and Why is music good?*

#### The impact of music passion

Music and being with others meant a lot to the participants: *“It's the music and meeting all my friends and being like a big family*”. Music Passion became the beginning of something new: *“Music Passion has meant a lot to me, because I think it was like the start of something new in my life”* and it was appreciated: *“It's the best thing that has happened in my life”*. One participant underlined the importance of trust: “*It's Music Passion that has helped me, it's the trust, it's nice people”*. The teaching method was important for some students: *“The teachers are great, they teach us in a good way, you feel appreciated”* and it was: “*Fun to learn new things about playing an instrument”.* All participants highlighted the performance as a great experience: “*And the show, what a great thing it was”* and many stated with joy, respect, and gratitude: “*Music Passion is a real school”*.

#### Why is music good?

One of the participants described what the music had done for him: “*Music has, well, what can I say, a way to get me on the train”.* Another said: *“Music is easier to use than anything else, that's why I love music”.* Several participants thought that music was fun and made them happy: *You become happy with music, music is fun and music means everything”* and one participant found that “*the good thing with music is that it is linked to other activities, for example dance”* and another described her feeling for the music and the way in which it affected her: *“I love music”, “I have a depression, music makes me think of something else, I calm down with music*”. Someone merely stated: “*I love music and that's the way it is”*.

### Staff reflections on participants' change

It was known that several participants had not worked well in a group before, but there had been no problems in Music Passion, which surprised the staff: “*They are always in a good mood when they are here”.* The staff had some ideas about why it had worked so well: “*Maybe it's because the rules were so clear”* or “*maybe the main thing is that you have a goal”.* They thought it might have something to do with the fact that Music Passion was an education, not only a leisure activity: “*Maybe it is because it is school activities”.* The importance of the teachers was mentioned: *“Could it be because here is someone you look up to, that they are real teachers?”* They also believed that the selection of participants affected the result: *“They have something in common, everyone is interested in music”*.

In the middle of the education the teachers noted that: “*The music activities have worked in a self-strengthening way and the students have become more independent both in the lessons and in their everyday lives. The students*’ *participation in decision-making has increased, they more often act on their own initiative*. This indicates good self-development.

The participants who completed the training formed a very heterogeneous group in terms of disabilities. Several have more extensive disabilities and can be described as multi-disabled, while others have a less extensive disability. Despite this, the Music Passion education achieved its purpose and goals. This is evidenced by educators, staff, relatives**,** and participants.

## Discussion

The overall purpose of the study was to evaluate the results of a 3-year education program with set goals for young adults with disabilities using a qualitative method.

The education and its goals were based on collaboration, which contributed to creating positive group cohesion. The participants interacted well with each other and enjoyed being together. Shortly after the start of the intervention the participants were divided into two groups for part of the teaching based on their different functional levels. However, this did not prevent them from collaborating to achieve a common goal. For example, both groups were included in the choir and all participants worked together in the planning and preparation of the final performance each year. All the participants wanted to be involved in that work and participate in the end of year performance. The three performances were very successful, all students performed their roles very well, and the response of the audience was overwhelming. This confirmed that it is possible to create positive group cohesion even when the group was divided for some parts of the teaching.

All the participants learned something new during the training, such as the necessary skills to play an instrument, stand as a presenter on stage, play a role in front of an audience, sing together or solo, dance or accompany an entire song on drums. The participants made optimal use of their resources and in the interviews, several expressed that they had discovered new sides of themselves and were surprised that they had been able to learn so much. For the last performance after the three-year education, several of the participants were also involved in writing the script.

Attendance at the lessons was very high, which can be assumed to be due to the students being very motivated to participate in the training. This is something that was confirmed by comments from the staff and in the interviews with the participants. The participants experienced the teaching activities and tasks as stimulating, fun**,** and meaningful. It was significant that the work had a clear goal, which they looked forward to achieving and that they learned something new. Furthermore, they were part of a social context where they felt accepted and worked in collaboration. They not only learned something new but also created something, which was appreciated by those around them.

People with a cognitive disability and a lack of ability to express themselves clearly are dependent on an environment that has good sensitivity and empathy. It is not always easy to understand people with disabilities and it is often difficult for a person with disabilities to understand others or be understood. The participants in this study experienced difficulties when growing up, felt misunderstood, and had poor self-confidence, which they have in common with many persons with disabilities. The Music Passion education contributed to a change. During the training, the participants met empathetic people who had experience of people with disabilities and who could set clear boundaries and rules, which created a sense of security. Furthermore, they showed confidence in the participants' ability, understood the importance of adapting the requirements to each participant's daily mood, and putting words to feelings, which in turn resulted in the participants' feeling of being respected for who they are. This led to the possibility of discovering new sides of themselves and experiencing that they have resources. They thus gained an increasingly more positive self-image, greater self-confidence, and their mental health improved.

To use one's resources optimally, a person must be motivated and committed. There are several different theories about the importance of motivation for learning and how to strengthen it. One of them is the Self-determination theory (SDT), which differentiates between internal and external motivation (22, 23). Internal motivation may be seen as even more important than external motivation, where the latter relates to rewards of various kinds or is intended to satisfy the environment. Internal motivation grows when one does something because it is experienced as fun or rewarding, for example, playing the piano because it is stimulating and enjoyable, not merely to satisfy one's parents. However, external motivation is also important and can be seen in the meaning of affirmation for the person's values and identity. A balance between internal and external motivation is thus desirable. In the interviews, the Music Passion participants described finding an optimal balance between internal and external motivation. They let their interest guide their participation in the activities and experienced great joy in learning something new, felt that the learning had meaning and goals, while they received strong affirmation from the environment, such as the audience at the performances. This positively affected their identity development: “*I am someone who can”*.

In the interviews, several participants mentioned a traumatic childhood and adolescence, especially difficult experiences at school and in other social contexts, resulting in negative self-esteem, low self-confidence, and in some cases even social isolation. Some of them described personal inner changes in the form of a new view of themselves, increased self-confidence, reduced symptoms of mental illness, more positive self-esteem, and an increased ability to regulate their emotions. A few had also begun to reflect on themselves as a person, gaining greater insight, which indicates an ability to mentalize (21).

The reason for the positive results of the Music Passion education is complex and can be found in a variety of coincidental factors. However, it is clear both in the interviews with the participants and in the process, notes made by the staff that it was important that Music Passion is an education with a goal, the annual performance, to work towards. This meant that the participants experienced the various elements of the education as meaningful, which in turn is of great importance for both learning and mental health.

A secure, emotional attachment is a prerequisite for a person to be able to build trust in other people and develop optimally, based on their innate resources (31). It is also important for building a so-called self and developing positive self-esteem and self-confidence (19). In Music Passion, the participants became attached to the staff and fellow participants, while staff members became attached to the participants, making them feel safe, accepted, valuable, and appreciated. The staff believed in the participants' ability to learn and develop. In such an emotional climate, it is not surprising that the participants were able to use their resources.

Stern describes self-development in different phases and at about 2–6 months one develops the core self, while the subjective self develops at 7–15 months (19). He considered that the self can only be built in the relationship with another person. One can lose touch with one's own feelings, which Stern calls the subjective self, when one's own emotional experiences are pushed into the background and the expectations and demands of others come to the fore (19). When the social self becomes dominant over the subjective self, we lose what Stern calls the core self. It may make us feel empty, which in turn can lead to mental illness. People with disabilities are dependent on support from the environment, which requires mutual adaptation. It is not difficult to understand that the social self can sometimes be too dominant for people with disabilities, thus leading to the loss of the core self (19).

Jon Monsen has emphasized the importance of emotional awareness for mental health (32). The central psychological dimension of what we describe as emotional awareness is the ability to identify, harbor, experience, and express feelings. It is also essential to have a person with whom you can share the feelings. Emotions are a motivating driving force, where emotional experiences and the ability to express oneself are basic, which underlines the importance of emotional awareness and emotional communication. A person who has a cognitive disability with deficiencies in language development is at greater risk of not developing full emotional awareness, which in turn increases the risk of mental health problems.

Stern reflected on why music has such a positive impact on people (24). He emphasized the meaning of vitality affects, stating that vitality is a kind of manifestation of being alive and that “*we live impressions of vitality as we breathe air”*. Stern holds that music can communicate vital affects, which resonate in others. Vitality affects differs from the basic affects, which are biological reactions (33). Instead, the vitality affect is a special experience, which arises in the encounter with stimulating, dynamic movements (24). It is an intra subjective experience, which touches us on a deeper level of the psyche and is, like emotional attachment, an important part of the so-called intersubjective communication and implicit knowledge of others. Stern claims that vital affects are aroused in human beings in emotional interaction and communication.

Another person who tried to define the impact of music is Dorit Amir (34, 35). She examined music therapists’ and clients’ experiences of music therapy and described them in the form of meaningful moments of insight and transformation on an intrapersonal level, which occurred spontaneously and intuitively during a creative activity, especially a musical experience. These meaningful moments were also unexpected and unpredictable. The music shared by the music therapist and client triggers an inner movement within both, which is consistent with Stern’s description of the vitality affect, thus confirming the great potential of music (24).

Finally, the positive results of the Music Passion education are also related to time. Three years gave the participants an ample opportunity to get to know each other, create trust, and allowed the teachers to adapt the education to the participants by taking account of their need for slow learning and that new knowledge must be consolidated by many repetitions in accordance with the theory that “*repetition is the mother of learning”* (36). To ensure a positive result, it is therefore essential not to compromise on the quality requirements of an education. The result points to many important factors and conditions that must be included in order to achieve a positive result. Music Passion education shows that persons with disabilities can learn, develop, and change on a personal level, which in turn lead to health and well-being.

An intervention that lasts for a long time has both advantages and disadvantages. If the intervention is too short, there is a risk that results will not be achieved, while if it is very long, events can occur that interfere with the intervention, meaning that the relationship between the intervention and results can be questioned. In this study, the intervention lasted for a long time, which was necessary due to its purpose. Nevertheless, as the results were for the most part very concrete, making it easy to establish whether “the participants had learned something new” and as the findings were formulated by the participants themselves and confirmed by the teachers and care staff, we think that the results can be considered trustworthy.

A limitation is of course that a single study is not sufficient to generalize the result. However, the findings reveal several important factors for both learning and positive personal development in people with disabilities, which are vital to follow up and further investigate in future intervention studies.

## Conclusion

The music education achieved its purpose and goals as evidenced by participants, teachers, and staff. The study showed that persons with disabilities can learn, develop, and change on a personal level, which leads to health and well-being. In addition, it demonstrated that if people with disabilities are offered a proper education, they are more motivated to attend. This is important information for the on-going development of equality and inclusion in society for persons with disabilities.

## Data Availability

The raw data supporting the conclusions of this article will be made available by the authors, without undue reservation.
